# Supplementary material for: Biofunctionalized Decellularized Tissue-Engineered Heart Valve with Mesoporous Silica Nanoparticles for Controlled Release of VEGF and RunX2-siRNA against Calcification
Source: Bioengineering (Basel). 2023 Jul 20;10(7):859. doi: 10.3390/bioengineering10070859 (PMC10376836; doi:10.3390/bioengineering10070859)
Supplement: Supplementary file 1 [file bioengineering-10-00859-s001.zip › bioengineering-2469224-supplementary.pdf]

bioengineering

Biofunctionalized Decellularized Tissue-Engineered Heart Valve with Mesoporous Silica Nanoparticles for Controlled Release of VEGF and RunX2-siRNA against Calcification

Wenpeng Yu [1,†], Xiaowei Zhu [2,†], Jichun Liu [1,*] and Jianliang Zhou [1,*]

Supplementary informations

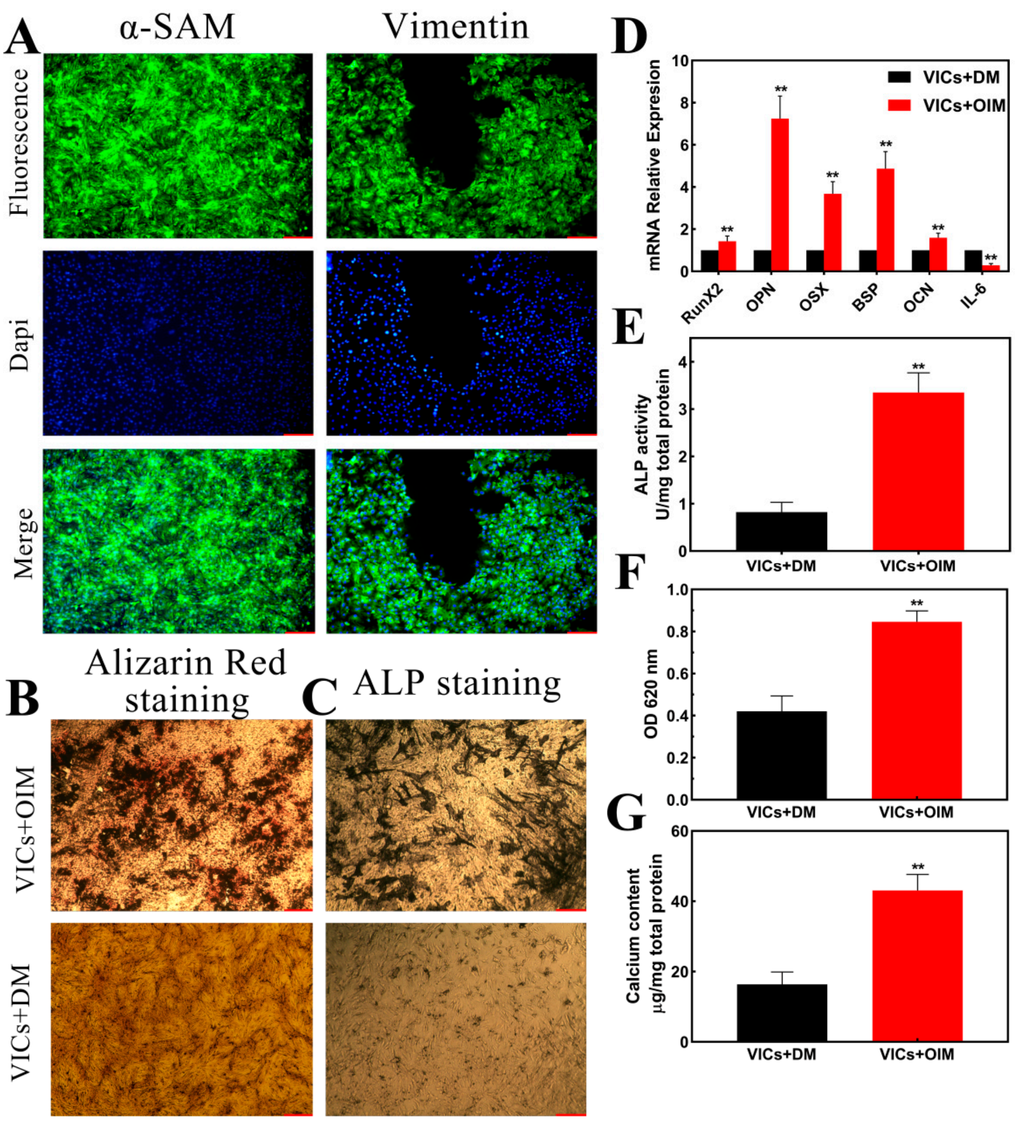

Figure S1. Evaluation of rat valvular interstitial cell calcification model. A. α-SAM and Vimentin immunofluorescence staining. B. Representative image of Alizarin red staining. C. Representative image of ALP staining. D. mRNA expression of related calcification genes. E. Quantitative analysis of ALP staining. F. Quantitative analysis of alizarin red staining. G. Determination of calcium content. Significant differences (* $p < 0.05$, ** $p < 0.01$), n = 3, scale bar = 20μm.

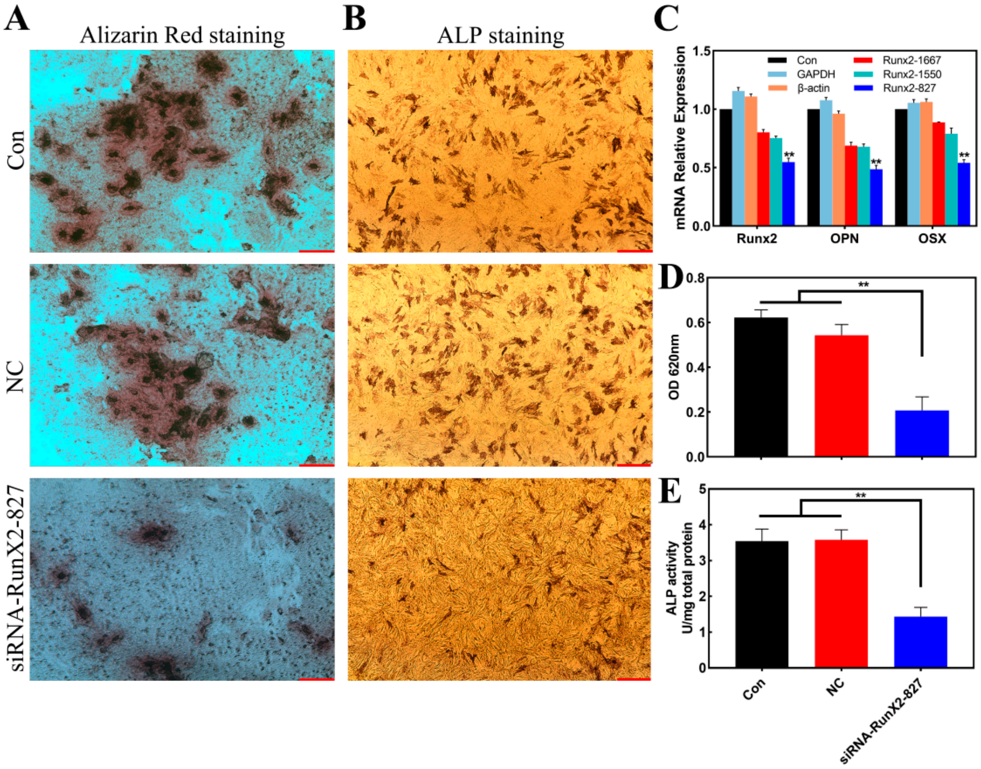

Figure S2. Anti-calcification of RunX2-siRNA. A. Representative image of Alizarin red staining. B. Representative image of ALP staining. C. mRNA expression of calcification genes associated with valve interstitial cells was detected after transfection with RunX2-siRNA. D. Quantitative analysis of alizarin red staining. E. Quantitative analysis of ALP staining. Significant differences (* $p < 0.05$, ** $p < 0.01$), n = 3, scale bar = 20μm.

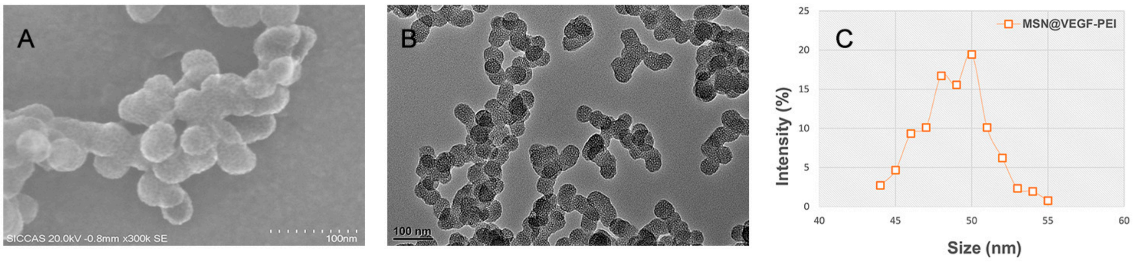

Figure S3. Characteristics of MSN@VEGF-PEI. A. SEM images of MSN@VEGF-PEI. B. TEM images of MSN@VEGF-PEI. C. Particle size analysis.

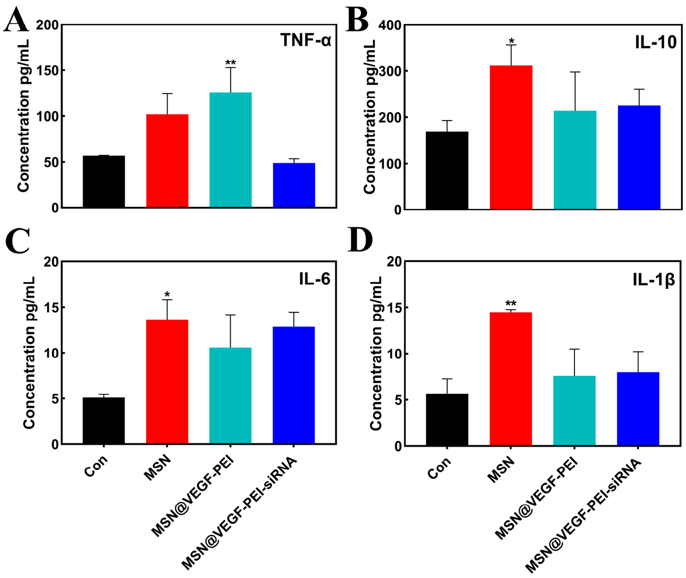

Figure S4. Release of inflammatory factors. A. TNF-α. B. IL-10. C. IL-6. D. IL-1β.Significant differences (* $p < 0.05$, ** $p < 0.01$), n = 3.

Table S1. Primer used in this study.

**Gene and primer direction**		**Sequence(5′ to 3′)**
**Rat GAPDH**	Forward	**GGAATCCACTGGCGTCTTCA**
	Reverse	**GGTTCACGCCCATCACAAAC**
**Rat RunX2**	Forward	**TCTTCCCAAAGCCAGAGCG**
	Reverse	**TGCCATTCGAGGTGGTCG**
**Rat OPN**	Forward	**CCAAGCGTGGAAACACACAGCC**
	Reverse	**GGCTTTGGAACTCGCCTGACTG**
**Rat OSX**	Forward	**GCCTACTTACCCGTCTGACTTT**
	Reverse	**GCCCACTATTGCCAACTGC**
**Rat BSP**	Forward	**CACTG GAGCCAATGCAGAAGA**
	Reverse	**TGGTGGGGTTG TAGGTTCAAA**
**Rat OCN**	Forward	**GCCCTGACTGCATTCTGCCTCT**
	Reverse	**TCACCACCTTACTGCCCTCCTG**
**Rat IL-6**	Forward	**CCTTCTTGGGACTGATGT**
	Reverse	**ACTGGTCTGTTGTGGGTG**

Abbreviations: GAPDH, glyceraldehyde-3-phosphate dehydrogenase; RunX2, core binding factor α1; OPN, osteopontin; OSX, osterix; BSP, bone sialoprotein; OCN, osteocalcin; IL-6, interleukin-6.
